# Advanced Melanoma Management: A Case Report of Pembrolizumab Use in a Haemodialysis Patient

**DOI:** 10.7759/cureus.78698

**Published:** 2025-02-07

**Authors:** Carina Teixeira, Rúben Costa, Miguel Silva, Maria João Ribeiro, Isabel Sousa

**Affiliations:** 1 Oncology, Unidade Local de Saúde de São João, Porto, PRT; 2 Dermatology, Unidade Local de Saúde de São João, Porto, PRT

**Keywords:** advanced melanoma, end-stage renal disease (esrd), haemodialysis, immunotherapy, pembrolizumab

## Abstract

Patients with end-stage renal disease are a challenge for oncologists. Despite the recognised benefit in the overall survival of pembrolizumab in patients with advanced melanoma, data on its safety and efficacy in dialysis patients are scarce. We present a clinical case of an 87-year-old woman on haemodialysis with advanced melanoma treated in the first line with pembrolizumab 200 mg every three weeks. She shows no significant adverse events and a good response to treatment. Although she underwent surgical treatment for two oligoprogressions, the patient maintained treatment with pembrolizumab, now demonstrating a response duration of 20 months. The age and comorbidities in this case, coupled with the lack of robust scientific evidence on the use of immunotherapy in dialysis patients, illustrate the daily complexity of therapeutic decisions in oncology.

## Introduction

According to Globocan 2022 data, cutaneous melanoma is the 17th most common cancer and the 22nd most lethal cancer worldwide [1]. The incidence of advanced melanoma is notably higher in older populations, with about 50% of diagnoses occurring in individuals aged 65 and older [2]. Older patients often present with more comorbidities, and before the development of immunotherapy and targeted therapies, they were frequently considered unfit for chemotherapy.

The current standard of care for the first-line treatment of unresectable stage III/IV melanoma includes immunotherapy - in monotherapy with anti-programmed cell death protein 1 (PD-1) inhibitors, combination therapy with anti-PD-1 and cytotoxic T-lymphocyte-associated antigen 4 (CTLA-4) inhibitors, or the combination of anti-PD-1 with anti-lymphocyte activation gene 3 (LAG-3) inhibitors - or targeted therapy with a combination of v-Raf murine sarcoma viral oncogene homolog B1 (BRAF) inhibitors and mitogen-activated protein kinase (MEK) inhibitors in the presence of a BRAF V600 mutation [2,3]. The selection between these treatment options depends on several factors, including prior systemic therapies (in neoadjuvant or adjuvant settings), BRAF mutation status, as well as the patient’s comorbidities and preferences. In cases of BRAF V600 mutation, evidence suggests that the sequence of immunotherapy followed by BRAF/MEK inhibitors in the event of disease progression results in improved outcomes compared to the sequence of BRAF/MEK inhibitors followed by immunotherapy [3].

The first immune checkpoint inhibitor proven to have a significant survival benefit in advanced melanoma was ipilimumab, an anti-CTLA-4 antibody. After that anti-PD-1 antibodies, such as nivolumab and pembrolizumab, also proven efficacy in advanced melanoma. In the phase 3 trial KEYNOTE 006, pembrolizumab showed an overall survival (OS) benefit demonstrating a median OS of 32.7 months versus 15.9 months for ipilimumab (hazard ratio 0.70; 95% CI, 0.58 to 0.83) [4]. Although pembrolizumab has shown safety and efficacy in most populations, including elderly patients [5], it has not been evaluated in clinical trials in patients with certain comorbidities, such as end-stage renal disease (ESRD). The prescribing information for pembrolizumab reports that its clearance is independent of renal function in cases of mild to moderate renal impairment. There are currently some case reports demonstrating the safety and efficacy of pembrolizumab in haemodialysed patients with advanced melanoma [6-8] and other cancers [7-10]. However, robust evidence for pembrolizumab in this specific population is still scarce. In light of this, we present a clinical case of an 81-year-old woman undergoing hemodialysis for ESRD due to autosomal dominant polycystic kidney disease (ADPKD), who received first-line palliative therapy with pembrolizumab, exhibiting good tolerance and favorable response.

## Case presentation

**Figure 1:**
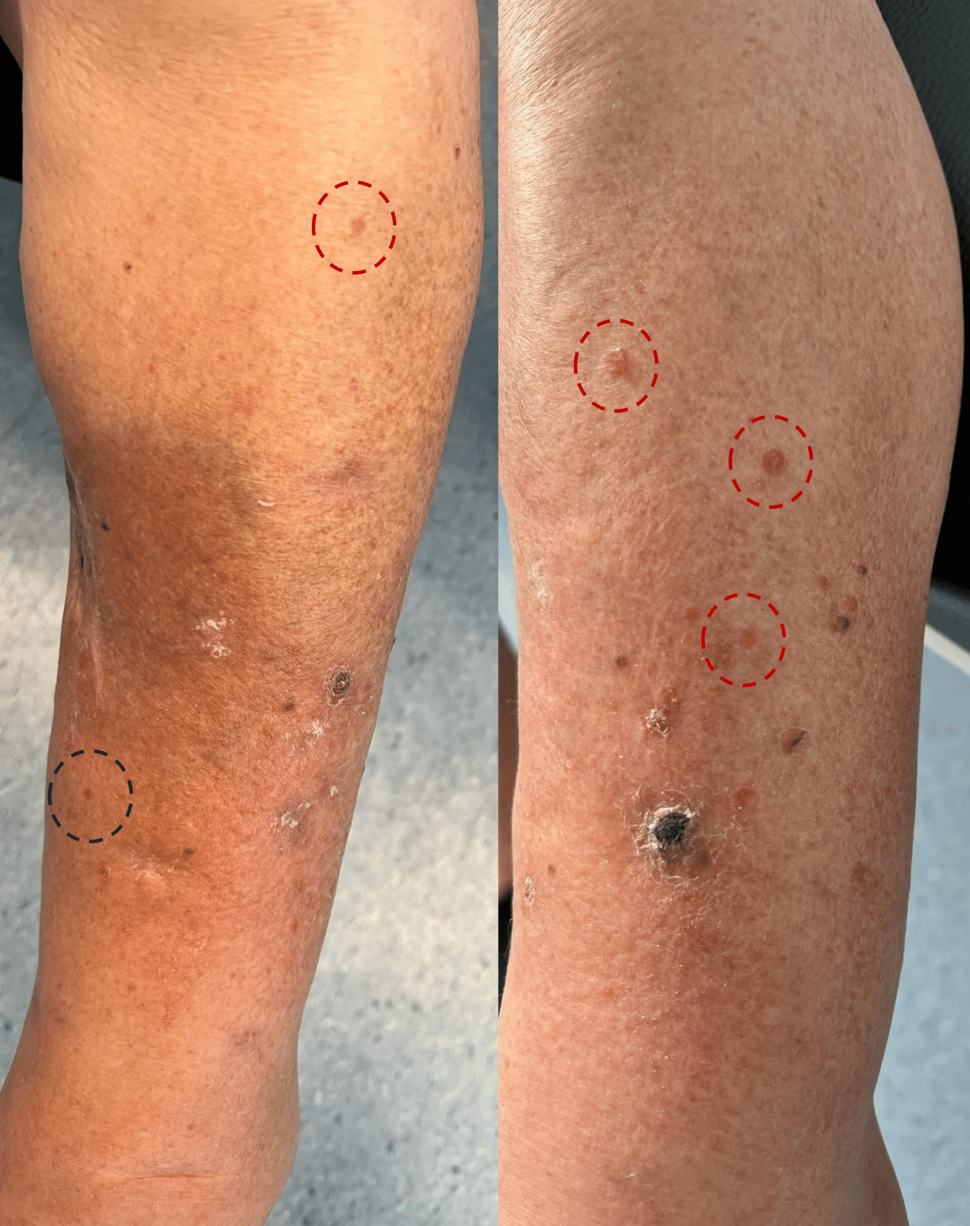
Nodular lesions compatible with melanoma metastases Multiple nodular lesions with a hard consistency, non-pigmented, and consistent with melanoma metastases are observed on the left leg, located more than 2 cm from the previous excision site, corresponding to in-transit metastases (highlighted by red circles). Additionally, a satellite lesion is identified in proximity to the prior excision site (marked by a blue circle).

An 87-year-old female patient with a history of hypertension, dyslipidemia, and atrial fibrillation, as well as ESRD on hemodialysis for four years due to ADPKD, first presented with a nodular lesion on the medial aspect of her left leg, measuring approximately 1.5 cm. The lesion had a three-month evolution and, on clinical examination, was firm, hyperpigmented, and exhibited irregular borders. The patient reported progressive enlargement of the lesion and recent onset of localized pruritus. Given these findings and a high clinical suspicion of melanoma, an excisional biopsy was performed. Histopathological analysis confirmed the diagnosis of melanoma (T3b, Breslow 3.1 mm, Clark level IV). Subsequently, margin widening and a sentinel lymph node biopsy were performed, both of which showed no evidence of disease, confirming a diagnosis of stage IIB melanoma. At the 10-month follow-up, the patient presented with a new nodular lesion, approximately 1 cm in diameter, located 2 cm below the site of the previously excised melanoma. The lesion was non-pigmented, painless, and exhibited a hardened consistency. An excisional biopsy later confirmed it to be a melanoma metastasis. Due to the patient's comorbidities, no adjuvant treatment was administered at the time. Five months later, the patient presented with multiple papular lesions surrounding the surgical scar on the left leg, raising suspicion of satellitosis and in-transit metastases (Figure 1).

**Figure 2:**
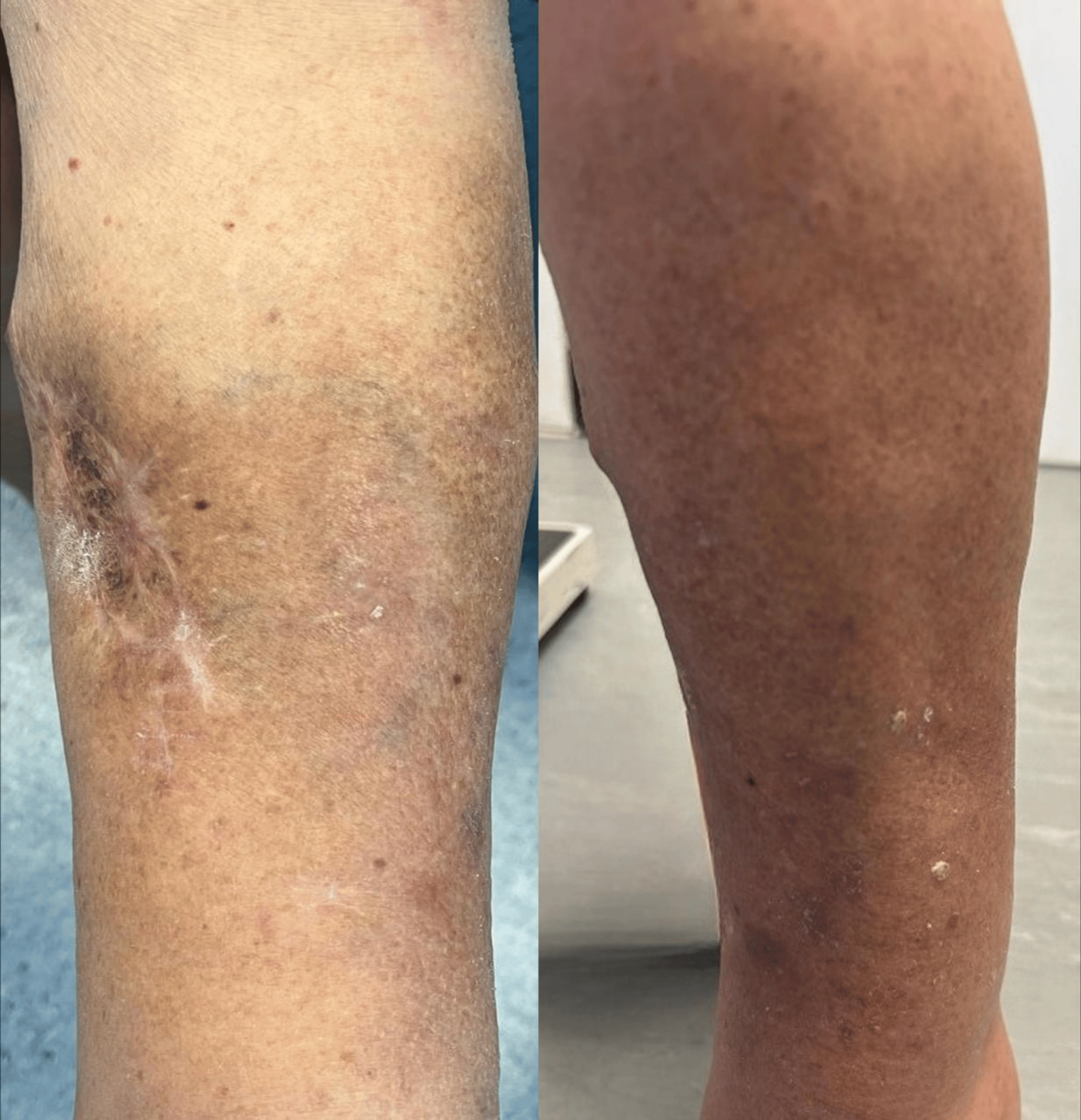
Clinical response after five cycles of treatment with pembrolizumab Significant response after five cycles of pembrolizumab, with the disappearance of satellite lesions and in-transit metastases in the left leg, without the emergence of new lesions.

An excisional biopsy of two lesions confirmed melanoma metastases, BRAF wild-type. Further imaging, including thoracoabdominal-pelvic computed tomography and brain magnetic resonance imaging, excluded additional metastases. The patient started treatment with pembrolizumab 200 mg every three weeks. After five cycles, a significant clinical response was observed, with a marked reduction in the cutaneous lesions (Figure 2) and no hypermetabolic lesions detected on positron emission tomography-computed tomography (PET-CT).

Following 16 cycles of treatment, a new nodular lesion appeared on the medial aspect of the lower third of the left leg, exhibiting hypermetabolic uptake on PET-CT. The lesion was excised, confirming melanoma metastasis, and the patient continued pembrolizumab. Five months later, after 24 cycles of treatment, PET-CT once again revealed hypermetabolic nodular thickening on the medial side of the left leg, indicating disease progression. Consequently, the patient underwent wide excision of the lesion. In the absence of further lesions, it was decided to maintain treatment with pembrolizumab. After 27 cycles of pembrolizumab, the patient continues to benefit from treatment with good tolerance and no toxicities. This represents approximately 20 months of therapy, during which the disease has remained controlled despite oligoprogressions, with no evidence of distant metastasis.

## Discussion

Immune checkpoint inhibitors (ICI), which enhance immune system function, have led to a notable improvement in the prognosis of patients with advanced melanoma [2]. Patients with ESRD have been excluded from trials of cancer therapy, including ICI, so estimating the safety and efficacy of these drugs is challenging [7]. In haemodialysed patients, the risk of drug accumulation and toxicity is a concern. Pembrolizumab is a selectively humanized IgG4 Kappa anti-PD1 monoclonal antibody that inhibits the PD-1 receptor on the surface of cytotoxic T lymphocytes. The clearance of pembrolizumab is not affected in patients with renal impairment, however, no guidelines have been established on the dose or timings for patients on dialysis [6]. An analysis of 98 case reports of ICI use in ESRD patients on dialysis showed a similar incidence of adverse events when compared to the normal population [7]. The effectiveness of ICIs in dialysis patients is also a matter of preoccupation. Firstly, the potential reduction in efficacy due to ultrafiltration. Studies involving antibodies of similar size to pembrolizumab have demonstrated that they are not dialyzable because of their high molecular weight. Consequently, it appears that the timing of dialysis does not impact the efficacy of pembrolizumab [6,10]. Additionally, patients with ESRD are immunocompromised, which may raise questions about the magnitude of the benefit of ICIs in this population. Anti-PD-1 checkpoint inhibitors work by blocking the PD-1 receptor on T cells, thereby enabling the activation of T cell-mediated antitumor responses. By disrupting this interaction, anti-PD-1 agents restore T cell activity, enhancing the immune system's ability to recognize and eliminate cancer cells, ultimately promoting antitumor immunity and inhibiting tumor growth. Therefore, the efficacy of pembrolizumab depends on the activation and effectiveness of the immune response. Despite the immunological challenges associated with ESRD, the analysis of reported cases [7] and our clinical case demonstrate that immunotherapy can be effective in these patients. 

To the best of our knowledge, there is no robust scientific evidence of survival outcomes and response rates for the use of ICI, in particular pembrolizumab, in dialysis patients. However, some case reports have shown good and sustained responses to immunotherapy in haemodialysed patients. In the previously mentioned publication involving 98 dialysis patients treated with ICIs, 29.6% exhibited complete or partial remission and 27.5% had stable disease, resulting in a disease control rate of 57.1% [7]. In terms of response duration, the longest duration identified in our research was in a patient with metastatic squamous non-small cell lung cancer, who experienced a partial response to pembrolizumab monotherapy for 45 months [10].

In our clinical case, the patient’s best response was a complete response. Despite receiving surgical treatment for two oligoprogressions, the advantages of pembrolizumab persisted, with no evidence of distant metastasis and a duration of response of approximately 20 months. No significant adverse events were observed.

## Conclusions

Immunotherapy has revolutionized the treatment landscape for advanced melanoma. This clinical case underscores the potential efficacy and tolerability of pembrolizumab in an elderly dialysis patient with advanced melanoma, highlighting the need for an individualized approach in this underrepresented population. As the aging population with multiple comorbidities continues to grow, bridging the gap between clinical trial data and real-world patients is essential. Given the exclusion of dialysis patients from clinical trials, real-life case reports play a crucial role in guiding treatment decisions. Further prospective studies are needed to better define the efficacy and safety of immune checkpoint inhibitors in this specific patient group.
